# Preterm birth, an unresolved issue

**DOI:** 10.1186/1742-4755-10-58

**Published:** 2013-11-15

**Authors:** Jose M Belizán, Justus Hofmeyr, Pierre Buekens, Natasha Salaria

**Affiliations:** 1 Instituto de Efectividad Clínica y Sanitaria (IECS), Buenos Aires, Argentina; 2 Effective Care Research Unit, Eastern Cape Department of Health, University of the Witwatersrand/Fort Hare, Eastern Cape, South Africa; 3 School of Public Health and Tropical Medicine, Tulane University, New Orleans, LA, USA; 4 Journal Development Editor, BioMed Central, London, UK

## Abstract

Premature birth is the world’s leading cause of neonatal mortality with worldwide estimates indicating 11.1% of all live births were preterm in 2010. Preterm birth rates are increasing in most countries with continual differences in survival rates amongst rich and poor countries. Preterm birth is currently an important unresolved global issue with research efforts focusing on uterine quiescence and activation, the ‘omics’ approaches and implementation science in order to reduce the incidence and increase survival rates of preterm babies. The journal Reproductive Health has published a supplement entitled Born Too Soon which addresses factors in the preconception and pregnancy period which may increase the risk of preterm birth and also outlines potential interventions which may reduce preterm birth rates and improve survival of preterm babies by as much as 84% annually. This is critical in order to achieve the Millennium Development Goal (MDG 4) for child survival by 2015 and beyond.

## 

Preterm birth is an unresolved global health issue. Globally it is the largest contributor to neonatal mortality and the second largest contributor to all under-5 mortality [1]. Preterm birth is also associated with long-term morbidity including developmental delay, cerebral palsy, retinopathy of prematurity, and hearing and vision problems [2,3]. Unlike other indicators of perinatal, maternal and infant health, there are not striking differences among rich and poor countries in the incidence of preterm birth [4]. Differences among poor and rich countries are mostly evident in the survival rate of preterm babies, which is mainly determined by the complexity of care that countries can provide [4,5]. The recent Global Burden of Disease study conducted by the Health Metrics Institute found that in 2010, for all ages and causes, complications from preterm births make up the 7^th^ greatest cause of disability-adjusted life years (DALYs) in developing countries. Whilst this ranking has decreased 4 places since 1990 there is a large continued disparity when compared directly with developed countries, where these complications make up the 35^th^ greatest cause, a decline from the 21^st^ in 1990 [6].

Paradoxically, some rich countries have higher rates of preterm birth than some very poor countries. There are many possible explanations for this anomaly. In rich countries there are several factors that can lead to preterm birth, such as advanced maternal age, In Vitro Fertilization techniques resulting in multiple pregnancies, high rates of caesarean sections, and the management of complicated pregnancies that may result in induction of a preterm birth. Unlike the majority of indicators during the reproductive period, preterm birth is a measure of age at birth rather than a disease, complication, or death. This measure of age requires good dating of the last menstrual period, which is a major constraint particularly for the poorest women in low-income countries who often receive late or no antenatal care. In some cultures, poor dating is related to poor observance of the calendar-year or the use of other calendars. Additionally for these women, use of early ultrasounds to better date their pregnancy is almost non-existent.

We are thus faced with this important and unresolved problem. For many years, intense research efforts have been focused on solving this problem, but still there is no solution. Preterm birth represents a common end point to a wide variety of clinical conditions, including spontaneous preterm birth with intact membranes or as a result of premature rupture of membranes, iatrogenic/medically induced preterm birth, multiple pregnancy preterm birth, foetal malformation/stillbirth, cervical incompetence, chorioamnionitis and self-induced preterm birth [7]. In some settings, high rates of hypertensive disorders of pregnancy contribute to the burden of iatrogenic preterm birth. Researchers should consider all of these situations in their approach. The study of uterine quiescence and activation has been the focus of much of this research but no solutions have been found. Many upcoming research initiatives are focused on the “omics” approaches: genomics, transcriptomics, proteomics and metabolomics [8]. Briefly, these disciplines aim to identifygenetic variations and the transcription of genetic factors to protein and metabolite production.

Another challenge is the fact that the few evidence-based interventions which could reduce the burden of preterm birth are often not used, especially in resource-limited settings. A relevant area of research relates to testing interventions that aim to increase the use of intervention of proven effectiveness: implementation science. Antenatal corticosteroids and Kangaroo Mother Care are attractive interventions that need to be tested in middle and low income countries in order to assess the best methods to implement them, and to measure the benefit in the survival of and future outcomes for preterm babies [9,10].

The journal Reproductive Health is very pleased to publish the supplement Born too Soon which underlines that preterm birth is an unfinished agenda for action and research [11]. This supplement is an outstanding effort involving many partners. It required major efforts from all those involved who are moved by the striking problem of preterm birth and who are devoted to achieving changes that can improve this unresolved situation. All contributors deserve recognition from those bearing the burden of being born too soon, and we hope that this venture will result in significant improvements. The supplement provides detailed information on every aspect of this subject, highlighting how critical the reduction of preterm births are for achieving progress on the Millennium Development Goal (MDG 4) for child survival by 2015 and beyond, and gives added value to maternal health (MDG 5) investments [1]. Estimates from this supplement show that, worldwide, an estimated 11.1% of all live births in 2010 were preterm (14.9 million babies born before 37 weeks of gestation), with preterm birth rates increasing in most countries [4]. Direct complications of preterm births account for one million deaths each year [4]. Papers in this supplement show that certain lifestyle factors in the preconception period may increase the risk for preterm birth, and therefore, that preconception care services for all women of reproductive age should address these risk factors by preventing adolescent pregnancy, preventing unintended pregnancies, promoting optimal birth spacing, optimizing pre-pregnancy weight and nutritional status including a multivitamin supplement containing folic acid, and ensuring that all adolescent girls have received complete vaccination [11]. This supplement also shows that the pregnancy period is a time when a woman can be reached with interventions aimed at reducing her risk of a preterm birth and potentially improving her health and the health of her unborn baby through a variety of mechanisms [9]. Papers from this supplement also describe effective neonatal care interventions that improve survival and later outcomes of preterm babies and point out that there are still around 50 million births outside of facilities where community interventions, including women’s groups, are promising strategies to reach these families who are often the poorest [5,9]. Finally, authors propose a series of selected interventions that, if applied universally, could save 84% or more than 921,000 lives annually, with antenatal corticosteroids and Kangaroo Mother Care having the highest impact [10].

This message is relevant to many stakeholders including scientists, clinicians, governments, and donors; a joint effort from all of whom could bring about these envisaged changes.

## Authors’ information

JMB is the editor-in-chief, JH and PB are members of the Editorial Board and NS is the Journal Development Editor of Reproductive Health Journal.
